# Inactivation of Indigenous Microorganisms and *Salmonella* in Korean Rice Cakes by In-Package Cold Plasma Treatment

**DOI:** 10.3390/ijerph18073360

**Published:** 2021-03-24

**Authors:** Joo Hyun Kang, Jaewoo Bai, Sea C. Min

**Affiliations:** Department of Food Science and Technology, Seoul Women’s University, 621, Hwarangro, Nowon-gu, Seoul 01797, Korea; as03226@naver.com (J.H.K.); jwbai@swu.ac.kr (J.B.)

**Keywords:** in-package cold plasma, Korean rice cake, indigenous microorganism, *Salmonella*, microbial inactivation mechanism

## Abstract

The antimicrobial effects of in-package cold plasma (CP) treatment on Korean rice cakes (KRC) were evaluated. The CP treatment (25 kV) inactivated indigenous mesophilic aerobic bacteria by 0.8–1.0 log CFU/g, irrespective of the position of KRC in the package. The addition of a shaking step during CP treatment increased the reduction in microbes by ~1 log CFU/g. The microbial inactivation efficiency increased significantly when the treatment time increased from 1 to 3 min. Microbial inactivation activity was highest for packages containing eight rice cakes. The optimized CP treatment achieved a 2.0 ± 0.1 log CFU/g reduction in indigenous bacteria. In addition, the optimum CP treatment inactivated indigenous yeast and molds and *Salmonella* in KRC by 1.7 ± 0.1 log CFU/g and 3.9 ± 0.3 log CFU/g, respectively. No significant changes in color and firmness were observed, and the surface temperature of KRC did not exceed 22 °C after CP treatment. Moreover, CP treatment damaged the cellular membrane of *Salmonella*, mainly by inducing lipid peroxidation. This study demonstrates the potential use of in-package CP treatment for the non-thermal microbial inactivation of KRC.

## 1. Introduction

Rice cakes are a widely consumed ready-to-eat food in Asia, particularly Korea, China, and Japan. With the recent increase in the consumption of home meal replacement products, the demand for Korean rice cakes (KRC) is increasing [1]. However, KRC has a short shelf-life due to the high possibility of microbial contamination during the manufacturing process [2,3]. To extend the shelf-life of KRC, microbial disinfection using sodium hypochlorite (NaOCl) solution or alcohol is widely used commercially. However, treatment with NaOCl generally creates chlorine-based chemicals, such as chloramines or trihalomethanes, which possess a risk of carcinogenesis [4,5]. Additionally, alcohol treatment generates an off-flavor [3]. To overcome these disadvantages, various methods have been evaluated for the inhibition of microorganisms in KRC, including altering the air composition in the packaging, adding organic acids and natural antibacterial agents (e.g., lactic acid and chitosan), and heating [2,6,7,8]. However, the aforementioned methods are still limited by the potential for microbial cross-contamination after treatment [9].

Cold plasma (CP) treatment has gained significant interest as a non-thermal, low-energy, versatile, and environmentally friendly technology [10]. CP treatment produces reactive oxygen species (ROS), such as ozone, superoxide anions, singlet oxygen, atomic oxygen, hydroxyl radicals, hydroperoxyl, alkoxyl, carbonate anion radicals, hydrogen peroxide, and peroxyl. It also generates reactive nitrogen species (RNS), such as nitrogen oxide, peroxynitrite, alkylperoxynitrite, and nitrogen dioxide radicals. In addition, hydrogen radicals, excited molecules, and charged particles are also generated by CP treatment. The type and concentration of ROS and RNS may vary depending on the plasma source, plasma generation gas, treatment voltage, treatment time, or humidity [11,12,13]. The generated reactive species inactivate microorganisms via damage to the cell membrane by etching or oxidation [11,14]. Previous studies have shown that CP treatment efficiently extends shelf-life by inactivating microorganisms in foods without adverse effects on food quality [15]. In particular, in-package CP treatment can be used to control microorganisms in food within packaging. Briefly, the packaged food is placed between two electrodes with high voltages to generate reactive species inside the packaging. As a result, the cross-contamination that occurs between the periods of post-microbial inactivation treatment and packaging does not take place if in-package CP treatment is applied [9]. Previously, Roh et al. (2019) have reported that in-package CP treatment (38.7 kV, 3.5 min) resulted in a 2 log CFU/cube reduction of *Salmonella* on chicken breasts packaged in a polyethylene terephthalate (PET) container [16]. In addition, Min et al. (2018) demonstrated the effective inhibition of *Salmonella* contamination in grape tomatoes packaged in PET commercial clamshell containers via in-package CP treatment (35 kV, 3 min) [17]. These studies have confirmed that in-package CP treatment is a promising new technology to inactivate microorganisms in a variety of foods, especially packaged ready-to-eat foods.

Various studies have focused on the inactivation of microorganisms in food using in-package CP treatment technology however, there are a lack of studies reporting the microbial inactivation efficiency of CP treatment on rice-based food, although few studies have presented the effect of CP treatment on functional and physical properties of rice flour and starch [18,19,20]. Thus, in this study, we aimed to develop an in-package atmospheric dielectric barrier discharge of CP treatment to inactivate natural mesophilic aerobic bacteria (hereafter, natural bacteria) and yeast and molds in packaged KRC and to control *Salmonella* contamination by evaluating the effects of various in-package CP treatment parameters, such as the position of KRC in the packaging, shaking during treatment, treatment duration, and the amount of KRC in packages on microbial inactivation. Furthermore, the effects of the in-package CP treatment on the color and texture of KRC were evaluated.

## 2. Materials and Methods

### 2.1. Preparation of KRC

A commercial KRC product was purchased from a local market. The major ingredients in KRC were rice (99%) and refined salt. Alcohol was used in preparation of the product. KRC samples were stored at 4 °C before use and all experiments were conducted within 2 days. The average weight of each KRC sample (diameter: 1.5 cm, length: 4 cm) was 8.0 ± 0.3 g. A PET container (width: 14 cm, length: 10 cm, and height: 3 cm; Dongyang D&P, Chilgok, Korea), sterilized with 70% ethanol, was used for KRC packaging. The container was capped with its lid tightly and the capped area was taped for hermetic sealing. KRC samples without pretreatment were used to evaluate the microbial inactivation efficiency of in-package CP treatment against natural bacteria and yeast and molds. To prepare *Salmonella*-contaminated KRC samples, KRC was boiled in water for 6 min, dried inside a biohazard hood (Hanbaek Co., Ltd., HB-402, Bucheon, Korea) for 1 h, and spiked with *Salmonella* inoculum.

### 2.2. Preparation of Salmonella Strains and Inoculation

Three *Salmonella* strains, *S. typhimurium* DT104, *S. montevideo* (CCARM 8052), and *S. enteritidis* (CCARM 8040), were used. *S. typhimurium* DT104 was provided by the Agricultural Biotechnology Culture Collection at Seoul National University (Seoul, Korea). *S. montevideo* (CCARM 8052) and *S. enteritidis* (CCARM 8040) were obtained from the antibiotic resistant strain bank (Culture Collection of Antibiotic-Resistant Microbes, Seoul Women’s University, Seoul, Korea). All strains were incubated at 37 °C for 24 h in tryptic soy broth agar (BD, Franklin Lakes, NJ, USA) and harvested. Then, each strain was washed three times using 0.1% (*w/w*) sterile peptone water (Difco, Becton and Dickinson, Detroit, MI, USA) with centrifugation (10,000 rpm for 2 min). The *Salmonella* inoculum (approximately 8 log CFU/mL) was prepared by mixing the same volume of each strain, followed by dilution. The *Salmonella* inoculum (50 μL, approximately 5 × 10^6^ CFU) was spiked on the upper side of KRC and spread evenly using a sterilized spreader. After drying on a sterilized table for 40 min, the other side was inoculated with *Salmonella* in the same way.

### 2.3. In-Package CP Treatment

**Figure 1:**
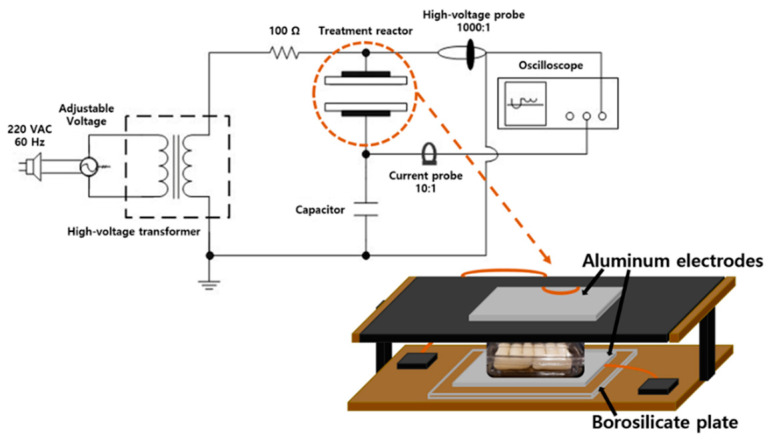
Schematic representation of in-package cold plasma treatment system used in this study.

The in-package CP treatment system (Figure 1) was comprised of a power generator, voltage transformer, and reactor with borosilicate placed between two aluminum electrodes (Kwang-lim Co. Ltd., Hwasung, Korea) located on the top and bottom [21].

**Figure 2:**
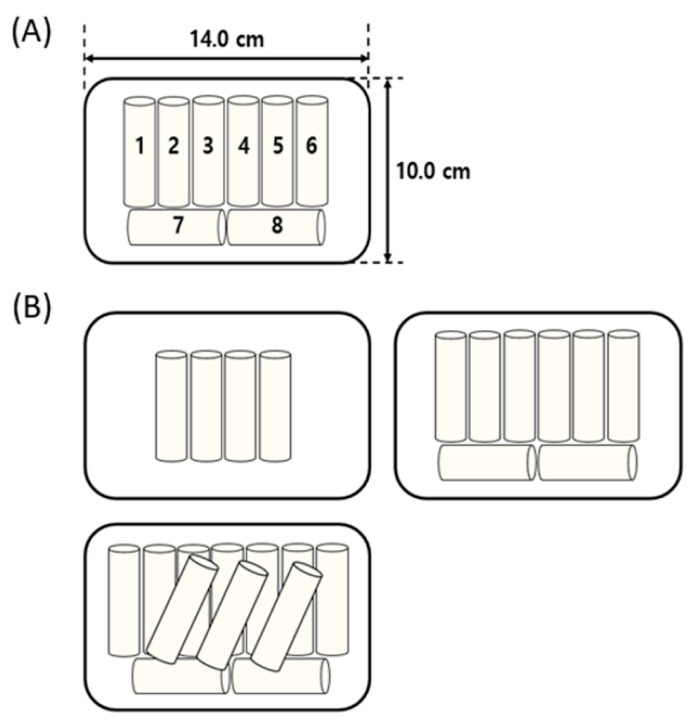
(**A**) Schematic representation of Korean rice cake positions in a polyethylene terephthalate (PET) container and (**B**) the cakes packaged in PET containers in different numbers (4, 8, and 12).

After placing a KRC-packaged PET container between the two electrodes, plasma was applied at 26 kV. This voltage was the maximum processing voltage not resulting in dielectric breakdown in KRC (data not shown). The distance between the upper surface of the packaging material and upper electrode was 0.5 cm. To investigate the effect of the location of KRC within the package on the inactivation of natural bacteria, KRC samples were placed in different configurations, as shown in Figure 2A, and treated with CP for 3 min without shaking. To evaluate the effect of a shaking step on the microbial inactivation efficiency, the 8-piece-KRC-containing PET package was shaken horizontally for 15 s (~0.5 hertz) every 45 s during the 3-min CP treatment period. CP treatment was performed for 1, 2, 3, 4, and 5 min with shaking using the 8-KRC package to investigate the effect of treatment time on microbial inactivation. To evaluate the effect of the number of KRCs per package on the inactivation of natural bacteria, packages including 4, 8, or 12 KRC pieces, as shown in Figure 2B, were subjected to CP treatment for 3 min with shaking. In all experiments, the package was kept unopened at 4 °C for 1 h after CP treatment. After incubation, the microorganisms were enumerated and the physicochemical properties of KRC were analyzed. An infrared thermometer (DT 44L; DIAS Infrared GmbH, Dresden, Germany) was used to measure the surface temperature of KRC before and after CP treatment.

### 2.4. Microbial Inactivation Test

To compare the effect of the position of KRC in the packaging on the microbial reduction, each KRC was removed one-by-one from a different location of the PET container after CP treatment. Then, each KRC was placed in a sterile bag (118 mL, Whirl-Pak, Write-On Bags; Nasco Co., Fort Atkinson, WI, USA) containing 10 mL of 0.1% sterile peptone water and was stomached for 3 min. In other experiments, all KRC samples (8) were put in a sterile bag containing 80 mL of 0.1% peptone water simultaneously and stomached for 3 min. After stomaching, each sample was 10-fold serially diluted in 0.1% peptone water and viable microorganisms were enumerated using a conventional plate count method. A Petrifilm aerobic count plate (3M^TM^, Seoul, Korea), xylose-lysine-deoxy chocolate agar (Difco/BD, Franklin Lakes, NJ, USA), and a Petrifilm yeast/mold plate (3M^TM^) were used to enumerate natural bacteria, *Salmonella*, and natural yeast and molds, respectively. Plates for natural bacteria and *Salmonella* were incubated at 37 °C for 24 h and plates for yeast/mold were incubated at 25 °C for 5 days prior to enumeration.

### 2.5. Investigation of Bacterial Inactivation Mechanism

To elucidate the mechanism by which in-package CP treatment results in *Salmonella* inactivation, changes in cell membrane integrity, lipid peroxidation, and intracellular ROS levels were measured after treatment. A *Salmonella* inoculum (9 log CFU/mL) was prepared and 3 mL of the inoculum was placed in a petri dish (35 mm diameter; SPL Life Science Co., Pocheon, Korea). Then, the *Salmonella* inoculum-containing petri dish was packaged in PET containers and treated with CP at 26 kV for 3 min. Propidium iodide (PI; Sigma-Aldrich, St. Louis, MO, USA), diphenyl-1-pyrenylphophine (DPPP; Sigma-Aldrich), and 5-(and -6)-chloromethyl-2′,7′-dichlorodihydrofluorescein diacetate (CM-H2DCFDA; Molecular Probes, Thermo Fisher Scientific, Eugene, OR, USA) were used to measure cell membrane integrity, lipid peroxidation, and intracellular ROS, respectively [22,23]. Briefly, PI (50 μM, final concentration), DPPP (2.9 μM, final concentration), and CM-H2DCFDA (5 μM, final concentration) were mixed with the treated or untreated *Salmonella* inoculum (1 mL) and incubated at 37 °C for 20, 10, and 15 min, respectively. After incubation, *Salmonella* cells were collected by centrifugation at 10,000× *g* for 10 min and washed twice using 0.1% peptone water. Fluorescence was measured at 351/380 nm (excitation/absorption) for PI uptake, 495/615 nm for the DPPP assay, and 495/520 nm for the CM-H2DCFDA assay using a spectrophotometer (SpectraMax 250; Molecular Devices, Sunnyvale, CA, USA). Fluorescence intensities were divided by the OD_600_ value for the quantitative comparison [23].

### 2.6. Chromaticity Measurement

The chromaticity of KRC was measured using a color-difference meter (Minolta Chroma Meter CR-400; Minolta Camera Co., Osaka, Japan). Briefly, the color-difference meter was calibrated with a white standard plate according to the manual. The L*, a*, and b* values were measured on three different spots of the KRC sample. The standard illuminate D65 and a 10° standard observer were used for measurement.

### 2.7. Texture Analysis

The texture of KRC was measured following the method described by Cheon et al. (2017). Briefly, the firmness (N) of KRC was determined using a texture analyzer (TA/XT2/25; Stable Micro Systems Co., Ltd., Surrey, UK). Measurements were obtained at three different positions for each KRC using a cylindrical probe with a diameter of 3 mm. The pre-test speed, test speed, post-test speed, and strain were 2.0 mm/s, 1.0 mm/s, 2.0 mm/s, and 40%, respectively.

### 2.8. Statistical Analysis

All experiments were independently repeated three times and, in each repetition, two measurements were made for each treatment (*n* = 6). One-way analysis of variance and Tukey’s multiple range tests were used to evaluate the differences between means. Statistical analyses were implemented in SPSS (ver. 24.0.0; IBM SPSS Inc., New York, NY, USA).

## 3. Results and Discussion

### 3.1. Effect of CP Treatment Time on Bacterial Inactivation

The initial concentration of natural bacteria in KRC before in-package CP treatment was 7.3 ± 0.4 log CFU/g. The microbial inactivation efficiency against natural bacteria on KRC was 0.8–1.0 log CFU/g, regardless of the position of KRC in the packaging (*p* > 0.05), indicating that CP was uniformly applied to each KRC sample (Table 1).

These results are consistent with those obtained by Roh et al. (2019), who reported that the inhibitory effect of in-package CP treatment on *Salmonella* on packaged chicken breasts during in-package CP treatment showed no significant difference with respect to the sample position [16]. These results suggest that the reactive species generated by the plasma treatment were generated uniformly inside the packaging and were evenly distributed to the empty spaces of the container.

**Figure 3:**
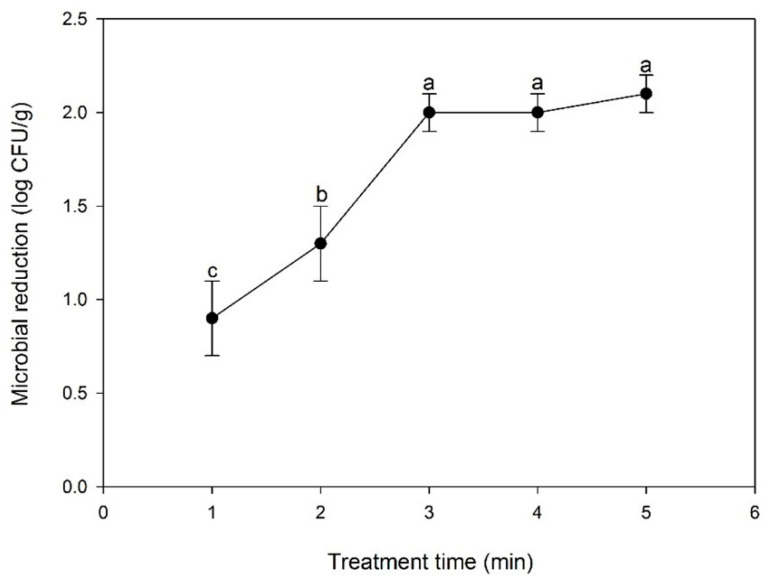
Effects of the in-package cold plasma treatment time on the inactivation of indigenous aerobic mesophilic bacteria in Korean rice cakes. The letters above the error bars indicate a significant difference (*p* < 0.05) between the test groups (*n* = 6).

The inactivation efficiency of in-package CP treatment against natural bacteria is shown in Figure 3. When CP treatment durations were 1, 2, 3, 4, and 5 min, the degrees of inhibition of natural bacteria in KRC were 0.9 ± 0.2, 1.3 ± 0.2, 1.9 ± 0.1, 2.0 ± 0.1, and 2.1 ± 0.1 log CFU/g, respectively. The inhibitory effects against natural bacteria increased significantly until 3 min. The enhanced antimicrobial activity may be due to increases in the concentrations of ROS and RNS inside the packaging as the CP treatment time increases [13]. Recently, Moutiq et al. (2020) reported that the inhibitory effects on aerobic mesophiles, psychrotorophs, and Enterobacteriaceae increased significantly in packaged chicken breasts as the in-package CP treatment time increases to from 1 to 5 min [24]. However, when the in-package CP treatment time increased from 3 to 5 min, no significant difference in the inactivation efficiency was observed (*p* > 0.05) (Figure 3). This result may reflect the limited amount of reactive species generated inside the packaging container [17]. Based on these results, an in-package CP treatment time of 3 min was identified as optimal and used for subsequent analyses.

The surface temperatures of KRC before and after CP treatment for 3 min were 17.9 ± 1.1 °C and 21.6 ± 0.7 °C, respectively. These results indicated that the bacterial inhibitory effect was not caused by a temperature change but by non-thermal CP treatment [25,26].

### 3.2. Effect of Shaking on CP Treatment Efficiency

The microbial inactivation efficiency was higher with the addition of the shaking step than without the shaking step. When the shaking step was added, a reduction in natural bacteria of 2.0 ± 0.2 log CFU/g in KRC was obtained, while a 1.0 ± 0.2 log CFU/g reduction was achieved without shaking. A beneficial effect of shaking on the microbial inactivation efficiency during in-package CP treatment has also been reported in previous studies. Min et al. (2018) reported that the efficiency of *Salmonella* inhibition by CP treatment increased by ~2.6 log CFU/tomato on packaged cherry tomatoes when shaking was applied [17]. In addition, Roh et al. (2019) showed that CP treatment with shaking increased the inhibitory effect on *Salmonella* on WPI-coated chicken breast by about 1 log CFU/sample [16]. These results clearly supported the increased inhibitory effect by the addition of the shaking step during CP treatment in KRC. The shaking step might result in the increased movement of reactive species and the degree of contaminated rice cake exposure to reactive species inside the package, thereby increasing the inactivation level of *Salmonella* [17,27]. Based on these results, we included a shaking step during in-package CP treatment in subsequent analyses.

### 3.3. Effect of KRC Number on CP Treatment Efficiency

The antimicrobial efficiency of in-package CP treatment according to the number of packaged KRC is shown in Table 2. For packages containing 4 and 8 KRCs, the numbers of natural bacteria were reduced by 1.9 ± 0.1 log CFU/g and 2.0 ± 0.1 log CFU/g, respectively, and no significant difference was observed between the two groups (*p* > 0.05). However, when the number of KRC was increased to 12, a 1.4 log CFU/g reduction was observed, which was lower than those in other groups (*p* < 0.05). This may be because the higher sample numbers reduced the empty space (the volume of atmospheric gas) in the package, decreasing the volume ratio of headspace to KRC (Table 2), which can result in the reduction in the reactive species generation [17]. Therefore, 8 KRCs per package were identified as optimal and used for further analyses of in-package CP treatment.

### 3.4. Evaluation of KRC Color and Texture

Generally, CP treatment does not have a substantial influence on food quality because the temperature change is minimal [28]. However, the generation of excessive reactive species can induce lesions on food surfaces by a process called “etching” and oxidize food components, resulting in the change in color [29]. As the color and texture of KRC are key determinants of consumer preference, it is important to evaluate quality changes following CP treatment [30,31]. Accordingly, color and texture changes of KRC under various CP treatment conditions were evaluated. No significant changes in the color and texture of KRC (*p* > 0.05) were observed after in-package CP treatment, regardless of the positions of KRC in the containers, shaking during CP treatment, treatment time, and the number of KRC in the containers (Tables S1–S4). This can be explained by the relatively low degree of etching and oxidation in KRC under the optimized CP treatment conditions [32]. In addition, CP can only affect the KRC surface based on its low penetration depth (i.e., 15 µm) [33] and therefore is not expected to have substantial effects on bulk properties, such as texture. Therefore, the in-package CP treatment applied in this study can effectively sterilize KRC without affecting the quality of KRC.

### 3.5. Control of Indigenous Yeast/Mold and Salmonella Contamination

Antimicrobial activity against indigenous yeast/mold and *Salmonella* contamination in KRC was evaluated under optimal CP treatment conditions. The initial concentrations of yeast/mold and *Salmonella* in KRC before CP treatment were 2.8 ± 0.1 and 6.2 ± 0.3 log CFU/g, respectively. In-package CP treatment significantly reduced viable yeast/mold and *Salmonella* to 1.7 ± 0.3 and 3.9 ± 0.3 log CFU/g, respectively. The lower microbial inactivation efficiency against yeast/mold cells than against *Salmonella* may be due to the rigidity of the fungal cell membrane, which contains chitin [11]. In the current study, the CP treatment for 3 min reduced the viable *Salmonella* number by 2.3 log CFU/g. The antimicrobial activity of CP treatment against *Salmonella* observed in this study is higher than that of a previously reported NaOCl (0.2 ppm) treatment (10 min), which inactivated *Salmonella* in KRC by 1.5 log CFU/g [34]. This demonstrates the potential of in-package CP treatment to enhance the microbiological safety of KRC. Moreover, CP treatment may replace the use of preservatives, such as alcohol, applied during the packaging process of KRC. Nonetheless, as CP treatment in the present state cannot eradicate microbial contamination in KRC, strategies such as a combination of CP with other treatments, including acid treatments, are suggested for investigation [35].

### 3.6. Salmonella Inactivation Mechanism

To investigate the mechanism underlying *Salmonella* inactivation by CP treatment, changes in cell membrane integrity, cell lipid peroxidation level, and intracellular ROS level were investigated (Table 3). PI assays are widely used to detect cell membrane damage; it can penetrate cells through pores in the cell membrane and stain intracellular nucleic acids [23]. The fluorescence level in the CP-treated group was higher than that of the untreated group, indicating the cell membrane of *Salmonella* was damaged by CP treatment (Table 3). DPPP, a reducing agent, is used to evaluate the degree of lipid peroxidation in cell membranes. Generally, lipids in the cell membrane react with hydrogen peroxide and oxidize DPPP to DPPP oxide (DPPP=O) [23]. As shown in Table 3, the lipid peroxidation level in CP-treated group was significantly higher than that in the untreated group (*p* < 0.05). This suggests that CP treatment induces lipid peroxidation in the *Salmonella* cell membrane, thereby resulting in cell death. When CM-H2DCF penetrates the cell membrane and is oxidized by intracellular ROS, it generates fluorescence. Thus, it quantitatively evaluates ROS produced inside the cells by measuring fluorescence [22]. Ziuzina, et al. (2015) reported that CP treatment increased intracellular ROS levels in Listeria monocytogenes [36]. However, in our study, no significant differences in fluorescence intensity were observed between the CP-treated and untreated groups (Table 3). We speculated that this difference among studies may be attributed to a difference in the mechanisms underlying the effects of plasma treatment against Gram-negative and Gram-positive bacteria. In Gram-negative bacteria, plasma-induced ROS damages cells mainly via membrane peroxidation, while ROS passes through the cell membrane and generates oxidative damage in intracellular components of Gram-positive bacteria [34]. As a result, the in-package CP treatment used in this study mainly controls *Salmonella* by damaging the cell membrane. Although further studies of DNA damage, protein damage, and other indirect damage by in-package CP treatment are needed in the near future [9], our current understanding of the mechanism underlying microbial inactivation by in-package CP treatment support the use of this method for the microbial inactivation of KRC.

## 4. Conclusions

The in-package CP treatment developed in this study uniformly inhibited indigenous mesophilic aerobic bacteria in KRC, regardless of the position of KRC in the package. The incorporation of a shaking step during in-package CP treatment increased the inhibitory effect. When the CP treatment time increased from 1 to 3 min, the inactivation efficiency of CP against indigenous bacteria in KRC was enhanced. In addition, the optimal microbial inactivation effect by CP was observed when the number of KRCs per package was eight. The in-package CP treatment effectively inactivated indigenous yeast/mold and *Salmonella* contamination with no significant changes in KRC color and texture. Further analyses revealed that CP treatment inactivated *Salmonella* mainly by cell membrane damage and lipid peroxidation. Additional studies, such as analyses of sensory properties, toxicity, and storage properties of processed foods and scale-up of treatment are required for the successful application of in-package CP treatment to KRC products. Nevertheless, the result of this study suggested that in-package CP treatment is a potentially effective non-thermal approach for the microbial inactivation of KRC.

## Figures and Tables

**Table 1:** Microbial inactivation efficiency of in-package cold plasma (CP) treatment against indigenous aerobic mesophilic bacteria at different positions in the container.

Sample Position (Numbers Designated in Figure 2)	Microbial Reduction (log CFU/g)
1	1.0 ± 0.3 ^a^
2	0.9 ± 0.3 ^a^
3	0.9 ± 0.1 ^a^
4	1.0 ± 0.3 ^a^
5	0.8 ± 0.1 ^a^
6	0.9 ± 0.0 ^a^
7	0.9 ± 0.1 ^a^
8	1.0 ± 0.4 ^a^

Results are expressed as means ± standard deviations (*n* = 6). Different lowercase letters indicate a significant difference based on Tukey’s test (*p* < 0.05).

**Table 2:** Effects of the number of Korean rice cakes on the microbial inactivation efficiency of in-package cold plasma (CP) treatment against indigenous aerobic mesophilic bacteria.

Number of Samples (Pieces)	Microbial Reduction (log CFU/g)
4	1.9 ± 0.1 ^a^
8	2.0 ± 0.1 ^a^
12	1.4 ± 0.1 ^b^

Results are expressed as means ± standard deviations (*n* = 6). Significant differences among groups in the same column are shown as lowercase letters based on Tukey’s test (*p* < 0.05).

**Table 3:** Changes in cell membrane integrity, lipid peroxidation, and intracellular reactive oxygen species (ROS) levels before and after in-package cold plasma (CP) treatments.

Treatment		Fluorescence	
	Cell Membrane Integrity	Cell Lipid Peroxidation	Intracellular ROS
Untreated	303.83 ± 64.48 ^b^	4282.68 ± 3.71 ^b^	1036.54 ± 29.25 ^a^
CP-treated	420.88 ± 6.61 ^a^	6393.00 ± 188.72 ^a^	1097.17 ± 1.74 ^a^

Results are presented as means ± standard deviations (*n* = 6). Significant differences among groups in the same column are indicated by lowercase letters based on the Tukey’s test (*p* < 0.05).

## Supplementary Materials

The following are available online at https://www.mdpi.com/1660-4601/18/7/3360/s1, Table S1: Effects of in-package cold plasma (CP) treatment on the color and firmness of rice cakes at various positions in the container, Table S2: Effects of shaking during in-package cold plasma (CP) treatment on the color and firmness of rice cakes, Table S3: Effects of the in-package cold plasma (CP) treatment time on the color and firmness of rice cakes, and Table S4: Effects of the number of Korean rice cake (KRC) in the container on the color and firmness of KRC after in-package cold plasma treatment.
