# Supplementary material for: Inactivation of Indigenous Microorganisms and Salmonella in Korean Rice Cakes by In-Package Cold Plasma Treatment
Source: Int J Environ Res Public Health. 2021 Mar 24;18(7):3360. doi: 10.3390/ijerph18073360 (PMC8036629; doi:10.3390/ijerph18073360)
Supplement: Supplementary file 1 [file ijerph-18-03360-s001.pdf]

(Supplementary information)

Inactivation of indigenous microorganisms and *Salmonella* in Korean rice cakes by in-package cold plasma treatment

Table S.1. Effects of in-package cold plasma (CP) treatment on the color and firmness of rice cakes at various positions in the container

Sample	Color						Firmness	
	Untreated			CP-treated			Untreated	CP-treated
	L*	***a****	***b****	***L****	***a****	***b****		
**1**	74.5±1.8a	-1.6±0.1a	7.4±1.7a	74.4±0.5a	1.5±0.1a	6.7±0.3a	41.5±4.6a	43.4±6.5a
**2**	74.2±1.1a	-1.6±0.1a	7.0±2.3a	73.6±1.0a	-1.6±0.1a	6.2±0.6a	44.4±5.5a	45.5±4.5a
**3**	74.3±1.9a	-1.6±0.1a	7.2± 1.4a	73.1±1.8a	-1.6±0.1a	6.1±0.4a	42.1±5.9a	45.7±4.8a
**4**	74.2±0.8a	-1.7±0.2a	7.0±1.2a	73.0±1.0a	-1.5±0.1a	5.9±0.4a	44.6±3.9a	43.4±8.3a
**5**	73.9±1.1a	-1.7±0.2a	7.0±1.1a	73.2±1.6a	-1.5±0.1a	6.6±0.4a	42.3±2.2a	42.7±0.7a
**6**	73.9±0.9a	-1.6±0.2a	7.2±1.3a	73.1±0.9a	-1.5±0.1a	6.3±0.7a	42.4±2.5a	43.6±2.5a
**7**	73.8±1.5a	-1.6±0.1a	7.3±2.0a	73.3±1.1a	-1.5±0.2a	6.2±1.2a	43.0±2.7a	44.5±3.0a
**8**	74.8±1.4a	-1.6±0.1a	6.8±2.0a	74.3±0.9a	-1.5±0.1a	6.8±1.0a	43.0±2.2a	45.2±6.0a

Results are expressed as means ± standard deviations (n = 6). Different lowercase letters in the same column indicate a significant difference based on Tukey's test ($P < 0.05$).

Table S.2. Effects of shaking during in-package cold plasma (CP) treatment on the color and firmness of rice cakes

**Treatments**		**Color**			**Firmness**
		L*	***a****	***b****	
**Untreated**		74.7±0.8a	-1.6±0.3a	7.3±0.4a	44.2±7.5a
**CP-treated**	Non-shaking	74.5±0.9a	-1.6±0.3a	7.3±0.4a	42.5±5.5a
	Shaking	74.5±0.8a	-1.6±0.2a	7.0±0.5a	42.8±6.2a

Results are expressed as means ± standard deviations (n = 6). Different same lowercase letters in the same column indicate a significant difference based on Tukey's test (P < 0.05).

Table S.3. Effects of the in-package cold plasma (CP) treatment time on the color and firmness of rice cakes

**Treatment time (min)**	**Color**			**Firmness**
	L*	***a****	***b****	
**0**	74.7±0.8a	-1.6±0.3a	7.2±0.4a	44.2±7.5a
**1**	74.7±1.2a	-1.3±0.1a	7.1±0.3a	43.4±5.3a
**2**	74.9±1.4a	-1.5±0.2a	6.7±0.5a	45.9±2.2a
**3**	74.5±0.8a	-1.6±0.2a	7.0±0.5a	42.8±6.2a
**4**	74.1±1.4a	-1.5±0.2a	6.8±0.3a	43.9±6.6a
**5**	74.0±1.0a	-1.6±0.2a	6.8±0.5a	42.2±5.1a

Results are expressed as means ± standard deviations (n = 6). Different same lowercase letters in the same column indicate a significant difference based on Tukey's test ($P < 0.05$).

Table S.4. Effects of the number of Korean rice cake (KRC) in the container on the color and firmness of KRC after in-package cold plasma treatment

**Treatment / number of KRC**		**Color**			**Firmness**
		L*	***a****	***b****	
**Untreated**		74.7±0.8a	-1.6±0.3a	7.2±0.4a	44.2±7.5a
	**4**	73.9±1.0a	-1.7±0.1a	6.9±0.3a	41.8±3.9a
**CP-treated**	**8**	74.5±0.8a	-1.6±0.2a	7.0±0.5a	42.8±6.2a
	**12**	74.0±1.1a	-1.4±0.1a	6.9±0.7a	42.2±4.8a

Results are expressed as means ± standard deviations (n = 6). Different same lowercase letters in the same column indicate a significant difference based on Tukey's test ($P < 0.05$).
